# Structural studies of a surface-entropy reduction mutant of O-GlcNAcase

**DOI:** 10.1107/S2059798318016595

**Published:** 2019-01-08

**Authors:** Alexandra Males, Gideon J. Davies

**Affiliations:** a Department of Chemistry, University of York, York YO10 5DD, England

**Keywords:** O-GlcNAc, O-GlcNAcase, neurodegeneration, surface-entropy reduction, crystallization

## Abstract

The enzyme O-GlcNAcase catalyses the removal of the O-GlcNAc co/post-translational modification in multicellular eukaryotes. The enzyme has become of acute interest given the intimate role of O-GlcNAcylation in tau modification and stability; small-molecular inhibitors of human O-GlcNAcase are under clinical assessment for the treatment of tauopathies. Given the importance of structure-based and mechanism-based inhibitor design for O-GlcNAcase, it was sought to test whether different crystal forms of the human enzyme could be achieved by surface mutagenesis. Guided by surface-entropy reduction, a Glu602Ala/Glu605Ala variant [on the Gly11–Gln396/Lys535–Tyr715 construct; Roth *et al.* (2017 ▸), *Nature Chem. Biol.* **13**, 610–612] was obtained which led to a new crystal form of the human enzyme. An increase in crystal contacts stabilized disordered regions of the protein, enabling 88% of the structure to be modelled; only 83% was possible for the wild-type construct. Although the binding of the C-terminus was consistent with the wild type, Lys713 in monomer *A* was bound in the −1 subsite of the symmetry-related monomer *A* and the active sites of the *B* monomers were vacant. The new crystal form presents an opportunity for enhanced soaking experiments that are essential to understanding the binding mechanism and substrate specificity of O-GlcNAcase.

## Abstract 2

The surface-entropy reduction method has been used to generate new crystal forms of human O-GlcNAcase.

## 1. Introduction   

The regulation of O-GlcNAc cycling on thousands of nuclear and cytoplasmic proteins is coordinated by two enzymes. O-GlcNAc transferase (OGT) catalyses the addition of GlcNAc, derived from UDP-GlcNAc, to serine and threonine residues, and O-GlcNAcase (OGA; CAZY database family GH84) cleaves O-GlcNAc (Torres & Hart, 1984 ▸; Holt & Hart, 1986 ▸; Kreppel *et al.*, 1997 ▸; Dong & Hart, 1994 ▸; Lubas *et al.*, 1997 ▸; Hart *et al.*, 2007 ▸). Two isoforms, OGA-L and OGA-S, are localized to the nucleus/cytoplasm (Comtesse *et al.*, 2001 ▸) and to the surface of lipid droplets, respectively. The reciprocal relationship between O-phosphorylation and O-glycosylation on the particular protein tau has been keenly studied in the context of neurodegeneration (Arnold *et al.*, 1996 ▸; Yuzwa *et al.*, 2008 ▸, 2014 ▸; Shen *et al.*, 2012 ▸; Griffith & Schmitz, 1995 ▸; Gao *et al.*, 2001 ▸; Liu *et al.*, 2004 ▸). In patients with Alzheimer’s disease, tau undergoes hyperphosphorylation, causing it to dissociate from microtubules and aggregate into paired helical filaments (PHF) and neurofibrillary tangles (NFTs) (Grundke-Iqbal *et al.*, 1986 ▸; Marotta *et al.*, 2015 ▸). O-GlcNAc cycling has also been implicated in tumorigenesis owing to its significant role in orchestrating a vast number of cellular processes, for example transcriptional and cytoskeletal regulation, cell signalling and division, and metabolism (Slawson & Hart, 2011 ▸).

**Figure 1:**
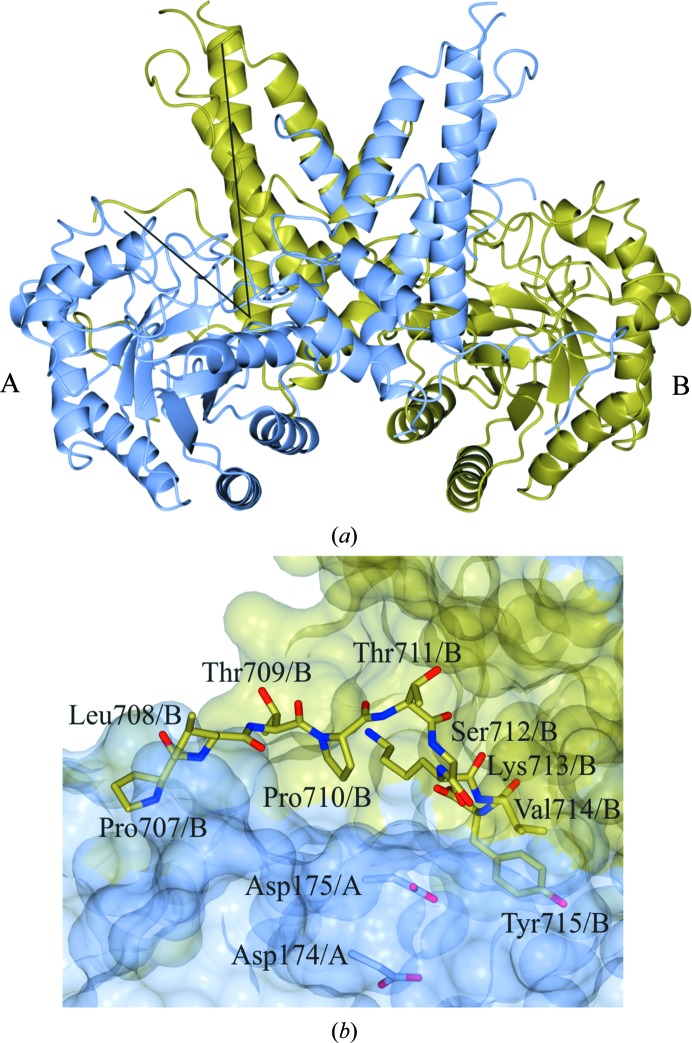
*Hs*OGA forms a dimer in solution and in the crystal structure. Monomer *A* is shown in blue and monomer *B* in gold. (*a*) One of the two active sites is indicated by intersecting black lines. (*b*) The C-terminal peptide, Pro707–Tyr715 in chain *B*, bound in the active site of monomer *A* with the catalytic residues, Asp174/*A* and Asp175/*A*, displayed.

Structural characterization of the human O-GlcNAcase orthologue (*Hs*OGA-L/*Hs*OGA) showed dimer formation both in solution and in the crystal where it has been shown to be promoted by helix-exchange (Roth *et al.*, 2017 ▸) in contrast to the non-helix-exchanged bacterial dimers (Dennis *et al.*, 2006 ▸; Schimpl *et al.*, 2010 ▸; Rao *et al.*, 2006 ▸). Composed of two ordered domains, the N-terminal glycoside hydrolase domain forms a (β/α)_8_ barrel (Li *et al.*, 2012 ▸), while the C-terminal stalk domain (Toleman *et al.*, 2004 ▸; He *et al.*, 2014 ▸) is composed of six α-helices. α17, consisting of Glu676–Pro694, undergoes a dimer ‘swap’ (Roth *et al.*, 2017 ▸), playing a structural role that contributes to dimerization (Li, Li, Lu *et al.*, 2017 ▸; Elsen *et al.*, 2017 ▸; Roth *et al.*, 2017 ▸). Located in a V-shaped cleft formed between the N-terminal domain of monomer *A* and the C-terminal domain of monomer *B* (Fig. 1 ▸ *a*) are the catalytic residues, Asp174 and Asp175, that initially act as a general acid and a general base through a two-step retaining mechanism (Macauley *et al.*, 2005 ▸). The terminal domain, which is not present in OGA-S, has a ‘pseudo’-histone acetyl­transferase activity but was not included in the crystallized construct in the three *Hs*OGA structures as it has a high degree of disorder.

The recognition mode for glycopeptide substrates has been explored by obtaining structures of the catalytically inactive Asp175Asn mutant of *Hs*OGA in complex with a variety of glycosylated peptides (Li, Li, Lu *et al.*, 2017 ▸; Li, Li, Hu *et al.*, 2017 ▸). The peptides bound in each structure can be segregated into two binding modes with forward or reverse orientations of the peptide (amino to carboxyl or carboxyl to amino, respectively) within the binding site. Initially, crystallographic peptide studies were conducted using an orthologue from the bacterium *Clostridium perfringens* (Schimpl *et al.*, 2012 ▸; Mariappa *et al.*, 2015 ▸) in complex with TAB1, lamin B1 and p53 glycosylated peptides. These peptides bound in the forward direction. Later, Li, Li, Hu *et al.* (2017 ▸) compared the same glycosylated peptides with *Hs*OGA and they were found to act in the same way. However, α-crystallin B and ELK1 bound in the reverse direction, strengthening the interest in determining how OGA selects its target (Li, Li, Hu *et al.*, 2017 ▸).

In the ‘apo’ structure, *Hs*OGA_11–396+535–715_ (thus named owing to the co-expression of two plasmids containing residues 11–396 and 535–715), which was published by Roth *et al.* (2017 ▸) and deposited as PDB entry 5m7r, the C-terminus of monomer *A* from residues Pro707 to Tyr715 can be traced in the reverse direction into the active-site groove of the symmetry-related molecule of monomer *B* (Fig. 1 ▸ *b*). When overlaid with the *Hs*OGA–p53 complex (PDB entry 5un8; Li, Li, Lu *et al.*, 2017 ▸), the position of the Tyr715 hydroxyl group lies directly on top of the O6 of O-GlcNAc.

To conduct biochemical/biophysical studies and rational drug design, complete and coherent structures are required. However, the published structures of *Hs*OGA have stretches of residues that are incomplete (Elsen *et al.*, 2017 ▸; Li, Li, Lu *et al.*, 2017 ▸; Li, Li, Hu *et al.*, 2017 ▸). For example, the structure described by Roth *et al.* (2017 ▸) has regions within the N-terminus (Glu11–Arg58 and Lys341–Thr370) and the C-terminus (Asp696–Pro706) that are not modelled in the structure. Expulsion of the C-terminus of *Hs*OGA_11–396+535–715_ from the active site is required before a competing compound can bind, therefore making it challenging to conduct crystal-soaking experiments; this is a problem for weak-binding compounds/inhibitors.

The work in this paper utilized surface-entropy reduction (SER) to enhance the structural characterization of *Hs*OGA and to contribute towards the hypothesis for the substrate-recognition mode of OGA, in which either the O-GlcNAc moiety or the peptide sequence is important for recognition. Using the *SERp* online server (Goldschmidt *et al.*, 2007 ▸), potential pairs of mutations were identified for *Hs*OGA. The rational design of mutating clusters of residues on a protein is a favourable strategy, with the aim of making the protein more susceptible to crystallization (Derewenda, 2004*a* ▸,*b* ▸; Derewenda & Vekilov, 2006 ▸; Cooper *et al.*, 2007 ▸). Surface-entropy reduction (SER) is a concept in which flexible, solvent-exposed residues, primarily lysine and glutamate, are mutated to alanine to reduce the entropic loss during the packing of the protein into a crystalline lattice (Longenecker *et al.*, 2001 ▸; Mateja *et al.*, 2002 ▸; Vekilov, 2003 ▸; Vekilov *et al.*, 2002 ▸).

Following the co-expression strategy of Roth *et al.* (2017 ▸) using pACYC-Duet-1 Gly11–Gln396 and pET-YSBLIC3C Lys535–Tyr715 plasmids, the successful pair of mutations was Glu602Ala/Glu605Ala (*Hs*OGA_E602AE605A_). Unlike *Hs*OGA_11–396+535–715_, the C-terminus of monomer *A* can be traced into the active site of the symmetry-related monomer *A*, with Lys713 binding in the position of O-GlcNAc. Furthermore, previously disordered loops had become ordered and could be built into the final model. Additionally, the activities and secondary-structure profiles of full-length *Hs*OGA (*Hs*OGA_FL_), *Hs*OGA_11–396+535–715_ and *Hs*OGA_E602AE605A_ were examined to verify the loss of the ‘pseudo-HAT’ domain and that the SER mutation did not alter the activity. The results showed that the SER mutant exhibited similar *K*_m_ values to the full-length enzyme, since the mutation is distant from the active site, highlighting the potential of SER variants for studying the structural and ligand-binding characteristics of *Hs*OGA.

## 2. Materials and methods   

### 2.1. Macromolecular production   

The cloning of the constructs has been described previously (Roth *et al.*, 2017 ▸). Homologous DNA for the Lys535–Tyr715 construct, in the vector pET-YSBL-LIC-3C, was mutated using a Q5 Site-Directed Mutagenesis Kit with Q5 Hot Start High-Fidelity DNA Polymerase. The forward primer, A GAT AGC gct AAA ATC gct GAA TGG, was designed to mutate the primary sequence A GAT AGC GAA AAA ATC GAA GAA; the reverse primer was TTA CCC TTG CAG TTA ACC GAA. NEB 5-alpha competent *Escherichia coli* cells were transformed with the Lys535–Tyr715 E602AE605A construct. The DNA was extracted and sequenced to verify the mutation.

The Gly11–Gln396 and Lys535–Tyr715 E602AE605A constructs in the vectors pACYCDUET-1 and pET-YSBL-LIC-3C, respectively, were co-transformed into *E. coli* BL21 (DE3) cells. Luria–Bertani broth was inoculated with a cell suspension and was incubated at 37°C until the OD_600_ reached 1.0. The cells were induced with 1 m*M* IPTG and incubated at 16°C for 20 h.

The purification of *Hs*OGA_E602AE605A_ followed the same purification protocol as that of *Hs*OGA_11–396+535–715_ described previously (Roth *et al.*, 2017 ▸; Supplementary Figs. S1*a* and S1*b*).

### 2.2. Crystallization   

*Hs*OGA_E602AE605A_ was initially crystallized by sitting-drop vapour diffusion at 15 mg ml^−1^ under condition E11 of the PACT *premier* screen from Molecular Dimensions (Newman *et al.*, 2005 ▸): 0.2 *M* sodium citrate tribasic, 20% PEG 3350. Further optimization of the conditions to 0.2 *M* sodium citrate tribasic pH 7.5, 17% polyethylene glycol 3350 in a 48-well MRC MAXI optimization plate improved the crystal shape. The total volume of the drop was 1 µl and the protein:reservoir solution ratio was 1:1; the total volume in the reservoir was 100 µl.

### 2.3. Data collection and processing   

Diffraction images were collected on beamline I04-1 at Diamond Light Source (DLS). After data collection, the diffraction images were integrated using the -3dii option in *xia*2 (Winter, 2010 ▸) and reintegrated using *AIMLESS* (Evans, 2006 ▸; Evans & Murshudov, 2013 ▸) from the *CCP*4 software suite (Winn *et al.*, 2011 ▸). Data-collection and processing statistics are given in Table 1 ▸.

### 2.4. Structure solution and refinement   

Molecular replacement against the coordinates of PDB entry 5m7r was conducted using *MOLREP* (Vagin & Teplyakov, 2010 ▸). Refinement of the model was conducted using multiple rounds of *REFMAC* (Murshudov *et al.*, 1997 ▸, 2011 ▸; Pannu *et al.*, 1998 ▸; Winn *et al.*, 2003 ▸; Vagin *et al.*, 2004 ▸; Nicholls *et al.*, 2012 ▸) and manual model building in *Coot* (Emsley *et al.*, 2010 ▸). Waters were added using Find Waters in *Coot* and validated. The data were processed to a resolution of 3.3 Å (Table 2 ▸).

### 2.5. Michaelis–Menten kinetics   

Michaelis–Menten kinetics were assayed using *Hs*OGA_FL_ and *Hs*OGA_11–396+535–715_ as positive controls against the mutant *Hs*OGA_E602AE605A_. In a 200 µl reaction volume, 50 n*M* protein solution and a serial dilution of the ligand 4-nitrophenyl *N*-acetyl-β-d-glucosaminide (*p*NP-GlcNAc) from 1500 to 11.7 µ*M* [dissolved in 2.5% DMSO (final concentration)] was added to PBS buffer at pH 7.4.

The reaction was monitored at 405 n*M* continuously using a Molecular Devices SpectraMax M5 spectrophotometer. The experiment, which was conducted at 25°C, was duplicated and each ligand concentration was repeated in triplicate.

*GraphPad Prism* v.5 was used to process the data, with nonlinear regression of Michaelian saturation curves. The initial velocities were calculated from the linear range of the reaction-progress curve. A standard curve of 4-nitrophenol was used to extract a molar extinction coefficient.

### 2.6. Circular-dichroism spectroscopy   

The protein samples were dialysed overnight into 25 m*M* sodium phosphate pH 8.0 and diluted to 0.1 mg ml^−1^. The spectra were recorded at 21°C in a QS 248 0.2 mm cuvette with 0.5 s per point and 78 s per read. The wavelength ranged from 195 to 320 nm. The background for each protein was measured immediately before the experiment in the same cuvette and values were taken as averages from triplicate reads.

## 3. Results and discussion   

### 3.1. Comparison of the mutant crystal structure with that of the wild type   

To incorporate protein molecules into a crystal, a thermodynamic cost is endured to bury hydrophobic residues into a constrained conformation and from the immobilization of the prevalent flexible hydrophilic side chains on the surface (Avbelj & Fele, 1998 ▸). Reducing the entropic shield can lead to an increase in the variety of conditions, morphologies and crystallographic space groups (Parthasarathy *et al.*, 2008 ▸; Kim *et al.*, 2005 ▸). Therefore, crystallization conditions were re-screened using the *Hs*OGA_E602AE605A_ variant; crystals were obtained in 17% polyethylene glycol 3350, 0.2 *M* sodium citrate pH 8.0. This is comparable to the wild-type crystallization condition of 0.1–0.2 *M* triammonium citrate pH 6.5–7.5, 16–24% PEG 3350.

In a different crystal form, flexible loops can become ordered by making backbone crystal contacts or adopting preferential conformations (Derewenda, 2004*a* ▸). The space group was *P*3_1_21, which is a lower symmetry group compared with *P*4_3_2_1_2 for the *Hs*OGA_11–396+535–715_ structure. The data statistics are presented in Tables 1 ▸ and 2 ▸.

Theoretically, SER should lead to an improvement in the resolution and hence the overall quality of the structure (Parthasarathy *et al.*, 2008 ▸). However, the structure was determined to a resolution of 3.3 Å, which was a lower resolution compared with the *Hs*OGA_11–396+535–715_ and catalytically inactive mutant structures, possibly owing to the thin-rod crystal morphology compared with the large trigonal bi­pyramid wild-type crystals.

**Figure 2:**
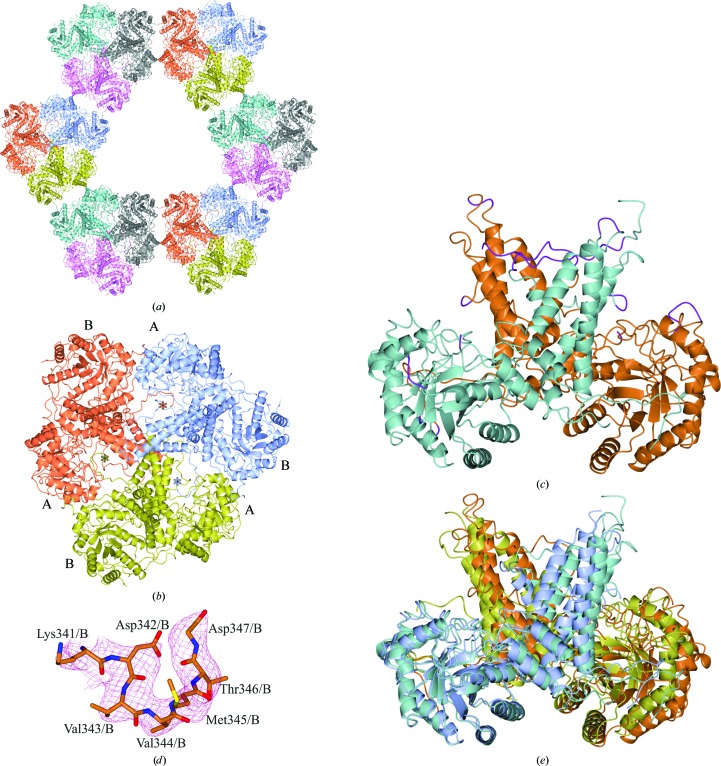
(*a*, *b*) Crystal symmetry of *Hs*OGA_E602AE605A_. (*a*) The connections made between the dimers show the trigonal solvent channel, with 50% solvent as calculated from the Matthews coefficient of 2.51 Å^3^ Da^−1^ (Kantardjieff & Rupp, 2003 ▸; Matthews, 1968 ▸). (*b*) Side view of the repeating trigonal dimers showing the linking C-terminus of monomer *A* binding into monomer *A* of the next dimer complex. The monomers are labelled *A* and *B*, with asterisks indicating the C-termini. (*c*, *d*) Disordered loop regions were stabilized in the new crystal form. Monomer *A* is shown in sea green and monomer *B* in brown. (*c*) The regions in purple were built into the *Hs*OGA_E602AE605A_ structure using PDB entry 5m7r as the template model and were missing from the wild-type structure. (*d*) Residues Lys341–Gly347 and the maximum-likelihood/σ_A_-weighted 2*F*_obs_ − *F*_calc_ map shown in red contoured at 0.12 e Å^−3^. (*e*) Overlap of the N-terminal monomers *A* from chain *A* for both *Hs*OGA_11–396+535–715_ (monomer *A*, blue; monomer *B*, gold) and *Hs*OGA_E602AE605A_ (monomer *A*, sea green; monomer *B*, brown) as calculated by *CCP*4*mg* *Superpose* models. The residue range selected for superposition was Arg59/*A*–His395/*A*.

A solvent channel can be observed through the crystal structure (Fig. 2 ▸ *a*). The dimers form a trigonal spring such that the C-terminus of monomer *A* binds into the active-site monomer *A* of a symmetry-related dimer, *etc.* (Figs. 2 ▸ *a* and 2 ▸ *b*). Owing to the different crystal contacts made on the surface of the protein, regions of high disorder could be built into the structure. 88% of the structure was complete, in comparison with 83% of the *Hs*OGA_11–396+535–715_ structure. The regions of highest disorder in both monomers were between Lys341 and Thr370, in addition to loops on the protein surface. After multiple rounds of refinement, the confidence for the inclusion of residues Lys341–Asp347 increased, enabling further visualization of the disordered region (Figs. 2 ▸ *c* and 2 ▸ *d*). The residues that were still disordered in monomers *A* and *B* were Gly11–Gly56, Ser348–Glu369, Lys535 and Pro707–Tyr715 for monomer *B* only. In the protein structures described by Elsen *et al.* (2017 ▸) and Li, Li, Lu *et al.* (2017 ▸) the residues Lys341–Asp371 in monomer *A* (PDB entry 5uhk) and Asn335–Val372 in monomer *A* (PDB entry 5tke), respectively, were also not observed.

When the N-terminal domains of monomer *A* from *Hs*OGA_E602AE605A_ and *Hs*OGA_11–396+535–715_ were overlapped, the r.m.s.d. of the N-terminal domains was 1.5 Å and the r.m.s.d. for the C-terminal domains was 7.1 Å, indicating a high degree of flexibility in the latter domain (Fig. 2 ▸ *e*).

**Figure 3:**
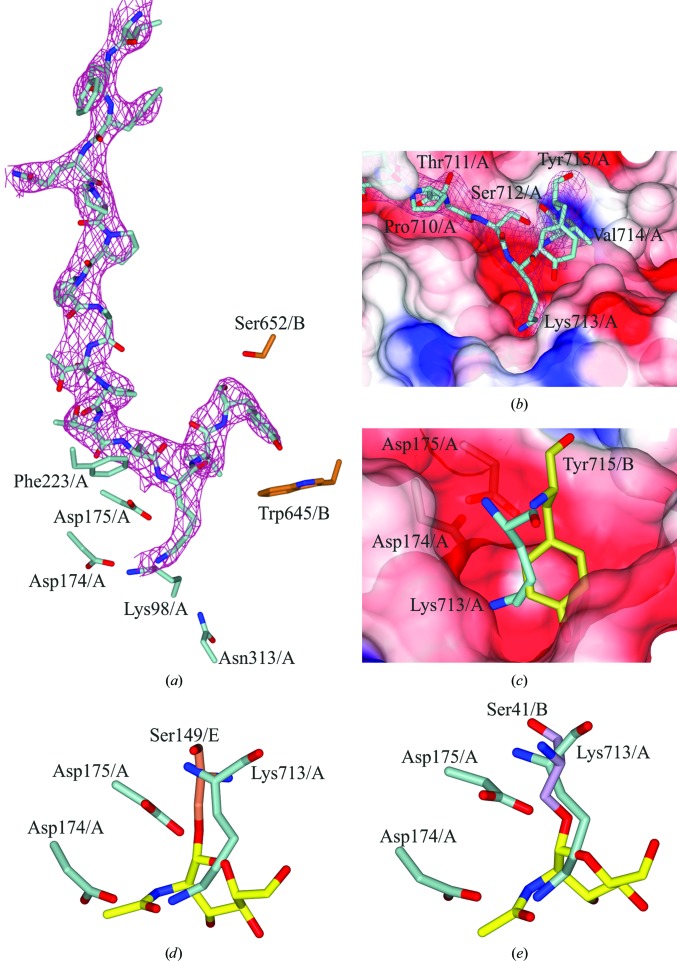
The C-terminus of monomer *A* binds into the active site of the symmetry-related monomer *A*. (*a*) Neighbouring residues in the active site of symmetry-related monomer *A* of *Hs*OGA_E602AE605A_, with the C-­terminal residues bound and the maximum-likelihood/σ_A_-weighted 2*F*_obs_ − *F*_calc_ map shown in red contoured at 0.17 e Å^−3^. (*b*) Surface representation of the active site with the C-terminus bound in a negatively charged pocket. (*c*) Overlay of Lys713/*A* from *Hs*OGA_E602AE605A_ and Tyr715/*B* from *Hs*OGA_11–396+535–715_ (in gold) in the binding pocket. (*d*) Overlay of the C-terminus of chain *A* of *Hs*OGA_E602AE605A_ and that of *Hs*OGA in complex with glycosylated p53 peptide (PDB entry 5un8), showing Ser149/*E* in coral. O-GlcNAc is shown in yellow. (*e*) Overlay of Lys713/*A* from *Hs*OGA_E602AE605A_ with *Hs*OGA in complex with glycosylated α-crystallin B (PDB entry 5vvv), showing Ser41/*B* in purple.

As mentioned, the C-terminus of monomer *A* was found to bind into the active site of a symmetry-related monomer *A*, aiding the formation of the new crystal packing (Figs. 2 ▸ *b* and 3 ▸ *a*). Initially, the residues of α17 interact with monomer *B* in a domain swap; the residues from Pro694 to Phe703 then bend back towards the residues of the respective monomer, with Gln704–Tyr715 binding into the active site (Figs. 3 ▸ *a* and 3 ▸ *b*). Electrostatic interactions between the C-terminus of monomer *A* and other residues of monomer *A*, *B* and a symmetry-related molecule *B* stabilize this formation. Pro707 of *Hs*OGA_E602AE605A_ has drastically moved position and faces in the opposite direction. There are three consecutive prolines that facilitate the change in direction. Hence, the C-terminus binds to the active site of the symmetry-related monomer *A* rather than monomer *B* (as in the wild type). The C-terminus of monomer *B* could not be built in from Pro707, indicating that it does not bind into an active site because of the crystal packing. In *Hs*OGA_11–396+535–715_, residues Asp696–Pro707 in monomer *A* are missing; therefore, the direction of the peptide is ambiguous. In the natural human sequence, the pseudo-histone acetyltransferase domain is connected to the stalk domain; therefore, binding of the C-terminus in the active site or alternatively C-termini disorder are possible artefacts of the removal of the HAT domain and of crystal packing.

The C-terminal residues Lys713/*A* and Tyr715/*A* hydrogen-bond to and make electrostatic interactions with residues of the active site (Fig. 3 ▸ *b*). Interestingly, the NZ atom of Lys713 is in the same position as the N2 atom of O-GlcNAc and is 2.97 Å away from the O^δ^ atom of Asp175/*A* on the symmetry-related monomer (Fig. 3 ▸ *d*). This pushes Tyr715 into the +2 subsite, where it interacts with the O^γ^ atom of Ser652/*B* of the symmetry-related monomer (Fig. 3 ▸ *a*). In comparison, the Tyr715/*A* hydroxyl group of the wild type lies above O6 of O-GlcNAc and hydrogen-bonds to the O^δ1^ atom of Asp285/*B* (Fig. 3 ▸ *c*).

In comparison to the crystal structure of *Hs*OGA in complex with α-crystallin B and ELK1 (PDB entries 5vvv and 5vvt, respectively; Li, Li, Lu *et al.*, 2017 ▸), the C-terminal residues are in the same reverse direction as the glycosyl­ated peptides (Fig. 3 ▸ *e*). This is in contrast to the structure containing a glycosylated p53 peptide shown in Fig. 3 ▸(*d*).

The density for all available *Hs*OGA peptide-complex structures supports the notion that OGA can bind peptides in both the forward and reverse directions. Comparison between the different peptide structures shows the versatility of the active site for different peptides.

### 3.2. Comparison of the constructs and mutants   

To ensure that the structural stabilization had not occurred owing to a change in the secondary structure of *Hs*OGA_E602AE605A_ and that the mutation had not affected the activity of the enzyme, the full-length protein (*Hs*OGA_FL_), *Hs*OGA_11–396+535–715_ and *Hs*OGA_E602AE605A_ underwent kinetic and secondary-structure analyses.

**Figure 4:**
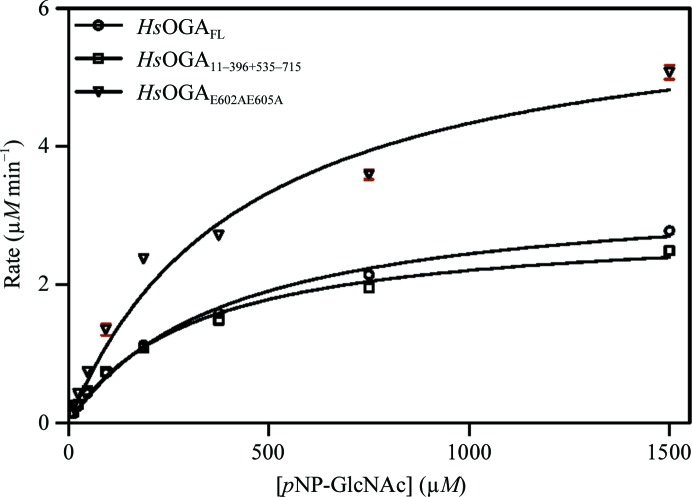
Michaelis–Menten curves for the kinetic assay of the *Hs*OGA mutant. *p*NP-GlcNAc was used as the substrate at concentrations up to seven times higher than the *K*_m_.

Comparison of the results for *Hs*OGA_FL_ and *Hs*OGA_11–396+535–715_ shows that the split construct has similar activity to the full-length variant, suggesting that co-expression of the two domains does not affect the activity (Fig. 4 ▸). Therefore, the ligand-binding data should be an accurate representation of binding to the full-length protein. When comparing HsOGA_11–396+535–715_ and HsOGA_E602AE605A_, the values are very similar. The *V*_max_ is higher, indicating an increase in the reaction rate (Table 3 ▸). The increase in *V*_max_ may be owing to a discrepancy in the enzyme concentration ([E]), as *V*_0_ is directly proportional to [E]. The similarity of *V*_max_ and *K*_m_ for the mutant and the wild type suggest that the mutation did not alter the substrate-binding affinity or the enzyme activity of the protein for its substrate.

**Figure 5:**
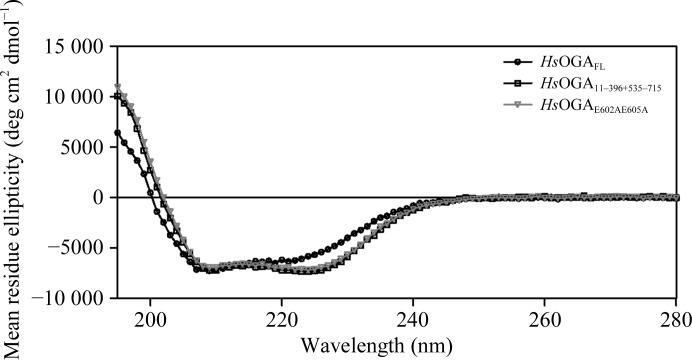
Circular-dichroism spectra for the different constructs. The experimental data in millidegree units were converted to mean residue ellipticity values with units of deg cm^2^ dmol^−1^ using the equation given in Ishtikhar *et al.* (2014 ▸).

The CD spectra show that all of the constructs are fully folded and the spectra for *Hs*OGA_11–396+535–715_ and *Hs*OGA_E602AE605A_ are very similar, suggesting that there is no change in the composition of the mutant (Fig. 5 ▸). The spectral profile of *Hs*OGA_FL_ differs from those of the split constructs in that it has a less pronounced minimum in the 222 nm region and an overall blue-shifted spectrum in the <210 nm region. This suggests that there could be a lower proportion of α-helical structure and/or higher structural disorder. This links to the inclusion of the pseudo-HAT domain that is connected to the C-terminal stalk domain. Since the structure of the human homologue of this domain is unknown owing to the inability of *Hs*OGA_FL_ to crystallize, the structure can only be inferred from the structural homologues from *Oceanicola granulosus* and *Streptomyces sviceus* (He *et al.*, 2014 ▸; Rao *et al.*, 2013 ▸). An estimate of the secondary-structure content of the proteins suggests a decrease of 6.4% in α-helical components and an increase of 3.7% in β-sheet components in *Hs*OGA_FL_ compared with *Hs*OGA_11–396+535–715_ (Supplementary Table S1). Homology modelling using crystal structures of the HAT domain from the bacterial homologue *O. granulosus* suggests a similar overall structure minus the acetyltransferase activity (Rao *et al.*, 2013 ▸). However, the data could be skewed by the inclusion of the linker region to this domain and the potential difference in the homology model structure. The secondary-structure contents of *Hs*OGA_11–396+535–715_ and *Hs*OGA_E602AE605A_ are consistent, further confirming that the mutation did not affect the overall structure.

## 4. Conclusions   

In this study, surface-entropy reduction has been utilized to produce further structural information on O-GlcNAcase by the incorporation of residues Ala57–Arg58, Lys341–Asp347, Thr370, Glu536, Cys596–Gly598, Gly674–Asp675 and Asp696–Pro707, an increase in the number of observed residues of 5%. Although the binding of the C-terminus to the active site may be an artefact of crystallization, it reveals further details regarding the substrate specificity of OGA, as peptides have been shown to bind in a bidirectional yet conserved conformation. The results described in this study present an opportunity for further investigation of the binding orientation of peptides within an SER-modified OGA enzyme. Given the progression of hOGA inhibitors into clinical trials, different surface mutants of the enzyme may afford new routes to drug development.

## Supplementary Material

PDB reference: surface-entropy reduction mutant of O-GlcNAcase, 6hki

Supplementary Figure and Table.. DOI: 10.1107/S2059798318016595/jc5018sup1.pdf

## Figures and Tables

**Table 1:** Data collection and processing Values in parentheses are for the outer shell.

Diffraction source	Beamline I04-1, DLS
Wavelength (Å)	0.9282
Temperature (K)	100
Detector	PILATUS 6M-F
Rotation range per image (°)	0.10
Total rotation range (°)	360
Exposure time per image (s)	0.040
Space group	*P*3_1_21
*a*, *b*, *c* (Å)	222.2, 222.2, 72.4
α, β, γ (°)	90.0, 90.0, 120.0
Resolution range (Å)	192.4–3.3 (3.5–3.3)
Total No. of reflections	523842 (75864)
No. of unique reflections	31103 (4440)
Completeness (%)	99.8 (98.6)
Multiplicity	16.8 (17.1)
〈*I*/σ(*I*)〉	7.3 (1.8)†
*R*_p.i.m._	0.11 (0.62)
CC_1/2_	0.99 (0.67)
Overall *B* factor from Wilson plot (Å^2^)	58.53

† The mean *I*/σ(*I*) in the outer shell fell below <2.0 at 3.4 Å resolution.

**Table 2:** Structure solution and refinement Values in parentheses are for the outer shell.

Resolution range (Å)	192.4–3.3 (3.5–3.3)
No. of reflections	31064
Final *R*_cryst_	0.17
Final *R*_free_	0.23
Cruickshank DPI	0.40
No. of non-H atoms	
Protein	7877
Water	1
Total	7878
R.m.s. deviations	
Bonds (Å)	0.010
Angles (°)	1.58
Average *B* factors (Å^2^)	
Protein	75
Water	16
Ramachandran plot	
Most favoured (%)	92
Allowed (%)	5

**Table 3:** Kinetics analysis comparing different *Hs*OGA constructs

Construct	*Hs*OGA_FL_	*Hs*OGA_11–396+575–715_	*Hs*OGA_E602AE605A_
*V*_max_ (µ*M* min^−1^)	2.96 ± 0.07	2.50 ± 0.06	4.62 ± 0.15
*K*_m_ (µ*M*)	298 ± 15	227 ± 13	230 ± 20
*k*_cat_ (min^−1^)	59.1 ± 1.4	49.9 ± 1.2	92.5 ± 3.1
*k*_cat_/*K*_m_ (min^−1^ µ*M* ^−1^)	0.198 ± 0.012	0.219 ± 0.013	0.401 ± 0.038
