# Supplementary material for: Structural studies of a surface-entropy reduction mutant of O-GlcNAcase
Source: Acta Crystallogr D Struct Biol. 2019 Jan 8;75(Pt 1):70–8. doi: 10.1107/S2059798318016595 (PMC6333286; doi:10.1107/S2059798318016595)
Supplement: Supplementary file 1 [file d-75-00070-sup1.pdf]

STRUCTURAL BIOLOGY

Volume 74 (2018)

Supporting information for article:

Structural studies of a surface-entropy reduction mutant of O-GlcNAcase

Alexandra Males and Gideon J. Davies

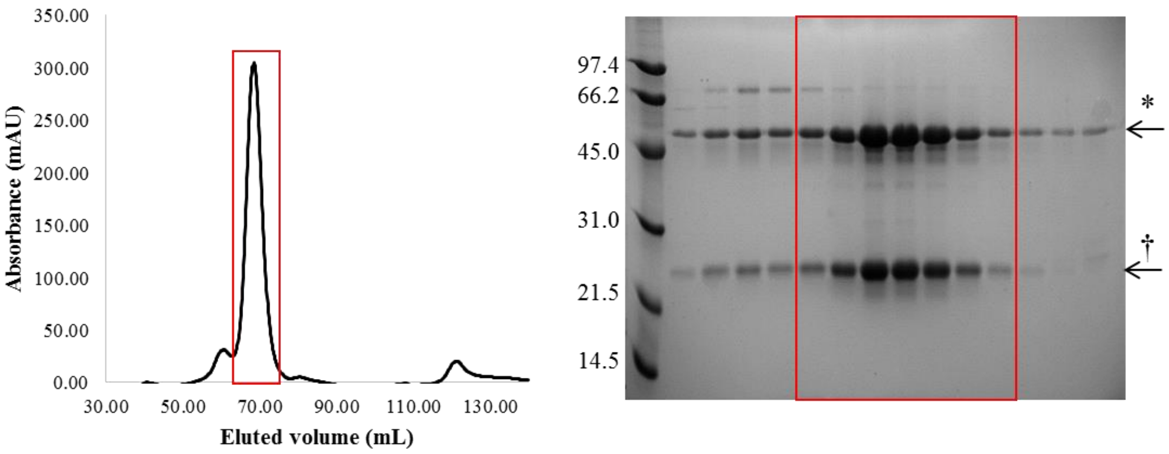

(*a*) (*b*)

Figure S1 Purification of *Hs*OGA$_{E602AE605A}$. The red boxes correspond to the identical fractions on the chromatogram and the gel that contain the purest sample, hence the fractions that were pooled. (*a*) Elution from a Superdex S200 column (GE Healthcare) resulted in a narrow peak after ~ 70 mL of elution buffer had passed through the column. (*b*) The resulting SDS-PAGE gel, 12%, showed two distinct bands for the two domains. * is the N-terminal catalytic domain that has a molecular weight of 46.6 kDa. † is the C-terminal stalk domain that has a molecular weight of 23.8 kDa. The protein ladder was Bio-Rad unstained low-range SDS-PAGE standards.

Figure S2

Table S1 Estimation of the secondary structure content of the constructs

Construct	*Hs*OGA$_{FL}$	*Hs*OGA$_{11\text{-}396+535\text{-}715}$	*Hs*OGA$_{E602AE605A}$
Predicted α (%)	9.89	16.33	16.33
Predicted β (%)	38.33	34.68	34.68
Max error	0.38	0.4	0.4
